# Supplementary material for: OpenMM 7: Rapid development of high performance algorithms for molecular dynamics
Source: PLoS Comput Biol. 2017 Jul 26;13(7):e1005659. doi: 10.1371/journal.pcbi.1005659 (PMC5549999; doi:10.1371/journal.pcbi.1005659)
Supplement: S1 Source Code — (ZIP) [file pcbi.1005659.s002.zip › OpenMM-7.0.1-Source/docs/api-python/generated/simtk.openmm.app.gromacstopfile.GromacsTopFile.html]

GromacsTopFile — OpenMM 7.0.1.dev-5e86c4f documentation


# GromacsTopFile¶

*class* `simtk.openmm.app.gromacstopfile.``GromacsTopFile`(*file*, *periodicBoxVectors=None*, *unitCellDimensions=None*, *includeDir=None*, *defines=None*)¶
:   GromacsTopFile parses a Gromacs top file and constructs a Topology and (optionally) an OpenMM System from it.

    `__init__`(*file*, *periodicBoxVectors=None*, *unitCellDimensions=None*, *includeDir=None*, *defines=None*)¶
    :   Load a top file.

        |  |  |
        | --- | --- |
        | Parameters: | - **file** (*str*) – the name of the file to load - **periodicBoxVectors** (*tuple of Vec3=None*) – the vectors defining the periodic box - **unitCellDimensions** (*Vec3=None*) – the dimensions of the crystallographic unit cell. For   non-rectangular unit cells, specify periodicBoxVectors instead. - **includeDir** (*string=None*) – A directory in which to look for other files included from the   top file. If not specified, we will attempt to locate a gromacs   installation on your system. When gromacs is installed in   /usr/local, this will resolve to /usr/local/gromacs/share/gromacs/top - **defines** (*dict={}*) – preprocessor definitions that should be predefined when parsing the file |

    Methods

    |  |  |
    | --- | --- |
    | `__init__`(file[, periodicBoxVectors, ...]) | Load a top file. |
    | `createSystem`([nonbondedMethod, ...]) | Construct an OpenMM System representing the topology described by this prmtop file. |

    `__delattr__`¶
    :   x.\_\_delattr\_\_(‘name’) <==> del x.name

    `__format__`()¶
    :   default object formatter

    `__getattribute__`¶
    :   x.\_\_getattribute\_\_(‘name’) <==> x.name

    `__hash__`¶

    `__reduce__`()¶
    :   helper for pickle

    `__reduce_ex__`()¶
    :   helper for pickle

    `__repr__`¶

    `__setattr__`¶
    :   x.\_\_setattr\_\_(‘name’, value) <==> x.name = value

    `__sizeof__`() → int¶
    :   size of object in memory, in bytes

    `__str__`¶

    `createSystem`(*nonbondedMethod=NoCutoff*, *nonbondedCutoff=Quantity(value=1.0*, *unit=nanometer)*, *constraints=None*, *rigidWater=True*, *implicitSolvent=None*, *soluteDielectric=1.0*, *solventDielectric=78.5*, *ewaldErrorTolerance=0.0005*, *removeCMMotion=True*, *hydrogenMass=None*)¶
    :   Construct an OpenMM System representing the topology described by this
        prmtop file.

        |  |  |
        | --- | --- |
        | Parameters: | - **nonbondedMethod** (*object=NoCutoff*) – The method to use for nonbonded interactions. Allowed values are   NoCutoff, CutoffNonPeriodic, CutoffPeriodic, Ewald, or PME. - **nonbondedCutoff** (*distance=1\*nanometer*) – The cutoff distance to use for nonbonded interactions - **constraints** (*object=None*) – Specifies which bonds and angles should be implemented with   constraints. Allowed values are None, HBonds, AllBonds, or HAngles. - **rigidWater** (*boolean=True*) – If true, water molecules will be fully rigid regardless of the value   passed for the constraints argument - **implicitSolvent** (*object=None*) – If not None, the implicit solvent model to use. The only allowed   value is OBC2. - **soluteDielectric** (*float=1.0*) – The solute dielectric constant to use in the implicit solvent model. - **solventDielectric** (*float=78.5*) – The solvent dielectric constant to use in the implicit solvent   model. - **ewaldErrorTolerance** (*float=0.0005*) – The error tolerance to use if nonbondedMethod is Ewald or PME. - **removeCMMotion** (*boolean=True*) – If true, a CMMotionRemover will be added to the System - **hydrogenMass** (*mass=None*) – The mass to use for hydrogen atoms bound to heavy atoms. Any mass   added to a hydrogen is subtracted from the heavy atom to keep their   total mass the same. |
        | Returns: | the newly created System |
        | Return type: | System |

High performance molecular simulation on GPUs


### Navigation

- Application Layer
  - Loaders and Setup
  - Representation and Manipulation
  - Simulation
  - Reporting Output
  - Extras
- Library Layer

©2015, Stanford University and the Authors.
|
Powered by Sphinx 1.3.1
& Alabaster 0.7.7
|
Page source
